# Flint corn silage management: influence of maturity stage, inoculation with *Lentilactobacillus buchneri*, and storage time on fermentation pattern, aerobic stability, and nutritional characteristics

**DOI:** 10.3389/fmicb.2023.1223717

**Published:** 2023-07-18

**Authors:** Luis G. Rossi, Marina E. B. Andrade, Carlos H. S. Rabelo, Gustavo R. Siqueira, Eduardo F. Vicente, Wilton L. Silva, Matheus M. Silva, Ricardo A. Reis

**Affiliations:** ^1^Department of Animal Science, São Paulo State University, Jaboticabal, SP, Brazil; ^2^Department of Plant Science, Federal University of Pelotas, Capão do Leão, RS, Brazil; ^3^São Paulo State Agency for Agribusiness Technology Alta Mogiana, Colina, SP, Brazil; ^4^Faculty of Science and Engineering, São Paulo State University, Tupã, SP, Brazil; ^5^Department of Animal Science, Federal University of Goiás, Goiânia, GO, Brazil

**Keywords:** corn silage, flint hybrid, maturity, starch digestion, storage length, *Lentilactobacillus buchneri*

## Abstract

**Introduction:**

High quality corn silage depends on factors such as corn type, stage of crop development at harvest time, fermentation time, in addition to use or not of inoculants. This study aimed to investigate the impact of maturity stage, bacterial inoculation, and storage time on fermentation, aerobic stability, and nutritional characteristics of flint corn silage and their implications for corn silage management.

**Methods:**

A flint corn hybrid was harvested very early, early, and medium (at 250, 300 and 350 g dry matter (DM)/kg as fed, respectively) and ensiled in mini-silos without (control) or with *Lentilactobacillus buchneri* CNCM I-4323 at 1 × 10^5^ cfu/g for 120, 240 and 360 d to investigate how these factors interact with each other.

**Results and discussion:**

There was only a small increase (7 g/kg starch; *p* = 0.003) in starch digestibility (starch-D) in the silages stored for 360 d when compared to that stored for 240 d, but with no difference for 120 d. Despite the reduced starch-D (526 vs. 694 g/kg starch; *p* < 0.001), silages produced from medium harvest had higher (*p* < 0.001) starch content (317 vs. 137 g/kg DM) and higher amount of digestible starch (169 vs. 98.5 g/kg DM; *p* < 0.001) compared to very early harvest. The 2-way interactions (inoculation × storage time and maturity × storage time) showed that inoculation of corn silage with *L. buchneri* increased (*p* < 0.001) the aerobic stability, and that more mature crop silage had higher aerobic stability (140 h; *p* = 0.036) than the others (118 and 48.5 h for those silages from very early and early harvest).

**Conclusion:**

The storage for a longer time (>120 d) with the goal of increasing silage digestibility did not occur. Harvesting whole-crop flint corn with 300 to 350 g/kg DM is desirable to have higher DM yield and starch accumulation. Inoculation with *L. buchneri* is recommended to preserve the silage against aerobic deterioration. This study has shown the importance of harvesting flint corn at the right time, and the need for inoculation with *L. buchneri* to ensure greater yield, starch accumulation, and silage preservation, if 120 days of storage are not exceeded.

## 1. Introduction

Most of the corn utilized for silage production in Brazil is flint type, which is recognized to have a high vitreousness that compromises starch digestion (starch-D) because the protein matrix surrounding the starch granules impairs ruminal microbial attachment. Ensiling has been proposed to increase starch-D (Saylor et al., 2021) and prolonging the storage time of silage was found to effectively improve starch-D (Da Silva et al., 2019). Such benefits are attributed to protein breakdown caused by the proteolytic activity of bacteria and plant enzymes (Junges et al., 2017). In this regard, a meta-analysis suggested that corn silage should be stored for at least 120 d to maximize starch utilization (Daniel et al., 2015).

As enhanced starch utilization is achieved by increasing storage time, this management is probably a feasible strategy to minimize the negative effects of the advances in plant maturity on starch-D. Indeed, starch-D is depressed by harvesting whole-crop corn with higher dry matter (DM) content (Der Bedrosian et al., 2012; Bueno et al., 2020), a physiological response attributed to the increased vitreousness of the corn grain (Philippeau and Michalet-Doreau, 1997). Conversely, despite the lowered herbage production and starch accumulation, it is known that harvesting whole-crop corn earlier than desirable (< 30% DM content) results in enhanced starch-D because the starch granules are more accessible to ruminal bacteria (Philippeau and Michalet-Doreau, 1997). Even though not desirable, livestock producers are forced to anticipate the harvest of whole-crop corn for silage production in some cases because of the lack of feed on the farm as a consequence of adverse climatic conditions that compromise feed production, lack of a feeding plan, and so on. Notably, many surveys carried out in Brazil have reported corn silages produced with DM contents lower than 30% (Daniel et al., 2019; Giombelli et al., 2019; Santos et al., 2020). In this case, where the harvest is anticipated, lower storage times of corn silage should be enough to ensure high starch-D once the starch granules are more susceptible to ruminal fermentation.

Moreover, silage inoculation has become increasingly common on farms, and in many cases, *Lentilactobacillus buchneri* is the bacterium used for improving the aerobic stability of silages. The most common dosage of *L. buchneri* used worldwide has been 1 × 10^5^ cfu/g of fresh forage (Bernardi et al., 2019; Arriola et al., 2021), because it has a good cost benefit. In addition to the reduction of silage spoilage in the presence of air, the inoculation of high-moisture corn (HMC) silage and rehydrated corn grain silage with *L. buchneri* at 1 × 10^5^ cfu/g of fresh forage was found to decrease the concentration of prolamin (Da Silva et al., 2018, 2019), and this effect was more evident in HMCs after 120 d of storage (Da Silva et al., 2019). The increased prolamin breakdown was suggested to be related to changes in the microbial community within the silo (Da Silva et al., 2019), probably directing toward those bacteria having higher proteolytic activity (Junges et al., 2017). The current study was designed to investigate as different ensiling strategies interact with each other, and then the dosage of *L. buchneri* tested (1 × 10^5^ cfu/g of fresh forage) was that most used on farm scale, which might be useful to increase starch-D in whole-crop flint corn silage.

We aimed to investigate the impact of maturity stage, bacterial inoculation, and storage time on fermentation, aerobic stability, and nutritional characteristics of flint corn silage and their implications for corn silage management. It was hypothesized that increasing storage time and using *L. buchneri* are feasible strategies to enhance starch-D of silages produced with 30–35% DM content at similar levels of that harvested very early and stored for less time.

## 2. Materials and methods

### 2.1. Ethics statement

All procedures adopted in this study were performed according to Ethical Principles in Animal Experimentation from the National Council for Animal Experiment Control (CONCEA) and were approved by the Ethics Committee on the Use of Animals (CEUA) from São Paulo State University (UNESP) at a regular meeting (Protocol No. 006764/17).

### 2.2. Crop harvest and ensiling procedure

A flint corn hybrid (2B 710 PW, Dow AgroSciences, São Paulo, SP, Brazil) was planted at a sowing density equivalent to 54,000 seeds/ha in 0.90-m rows in fields at São Paulo State University (at Jaboticabal, SP, Brazil: 21°150S, 48°180 W; altitude 615 m). One week prior to planting, herbicides (4 L Zapp®/ha and 0.5 L Select®/ha; Syngenta, Matão, SP, Brazil) and mineral oil (0.5 L/ha) were applied to the field. The sowing date was 10 November 2016, and the soil was classified as Haplustox. The fields were fertilized with 350 kg/ha of 8–28-16 (N–P–K) at planting. Thereafter, on 17 November 2016, an additional fertilizer, 300 kg/ha of 30–0-10 (N–P–K), was applied after a week of corn growth, and a further 350 kg/ha of urea was applied after 4 weeks of corn growth on 7 December 2016. Herbicides (3 L Zapp®/ha and 2 L Atrazine®/ha; Syngenta, Matão, SP, Brazil) and insecticide (0.25 L Engeo Pleno S®/ha; Syngenta, Matão, SP, Brazil) were applied after 4 weeks of corn growth on 5 December 2016. Fungicide (0.5 L Priori Xtra®/ha; Syngenta, Matão, SP, Brazil), insecticide (0.15 L Ampligo®/ha; Syngenta, Matão, SP, Brazil) and mineral oil (0.5 L/ha) were also applied after 5 weeks of corn growth on 12 December 2016. The climate where the corn was cultivated is classified as ‘Aw’ (Rolim et al., 2007) and characterized as tropical with a wet summer season and dry winter season.

On 31 January 2017, whole-crop corn (83-days growth) was randomly harvested in different locations in the field at 246 g of whole-plant DM/kg as fed (called ‘very early harvest at 250 g/kg DM’) at a stubble height of 20 cm using a pull-type New Pecus forage harvester (Nogueira, São João da Boa Vista, SP, Brazil). Forage was cut to 10 mm, and kernels were processed. The same process was repeated on 9 February 2017 (92-days growth) and 16 February 2017 (99-days growth) when the corn forage had 306 g/kg DM (called ‘early harvest at 300 g/kg DM’) and 353 g/kg DM (called ‘medium harvest at 350 g/kg DM’), respectively. To determine the DM yield and percentage of corn grains in each maturity stage, five points in the field were sampled for number of plants per linear meter, weight of plants and grains, and DM content. Thereafter, for each maturity stage in which corn plant was harvested, one pile of corn forage for each silo was randomly treated either with water (5 L/t; control) or with *Lentilactobacillus buchneri* (CNCM I-4323) at 1 × 10^5^ cfu/g of fresh forage (inoculated; Lallemand Animal Nutrition, Goiânia, GO, Brazil). The inoculant was dissolved in distilled water (5 L/t) and sprayed onto fresh forage during the filling of the silos.

Treated forages (3.21 ± 0.103 kg) were then placed into each mini-silo (*n* = 4). Forage packing was achieved by using wood sockets, and a final bulk density of 743 ± 23.9 kg fresh forage/m^3^ was obtained. PVC tubes (4.3 L) were used as mini-silos, and they were closed with plastic lids and sealed with adhesive tape. Mini-silos were stored in a barn at ambient temperature for 120, 240, and 360 d. One fresh sample of corn forage was collected from each silo during filling for chemical analysis. The same procedure was repeated when the silos were opened; both forage and silage samples were stored at −20°C. The DM recovery for each silo was calculated based on the initial and final weight and DM contents of the fresh forages and silages. Moreover, the recovery of digestible DM was calculated taking into account the initial and final weight of digestible DM, which was obtained by multiplying the dry weight of forage and silage placed inside each mini-silo by its respective *in vitro* DM digestibility (IVDMD).

### 2.3. Aerobic stability

Aerobic stability was determined by placing a silage sample (1.34 ± 0.133 kg) from each mini-silo in a plastic bucket of 5 L capacity and kept in a room at ambient temperature. The silage temperature was measured every half hour by using a datalogger (ESCORT Intelligent MINI; Escort Console, Buchanan, VA, United States) placed in the center of the mass for 10 d. The ambient temperature was also measured every half hour by two dataloggers placed near the buckets. Aerobic stability was defined as the number of hours that the silage temperature remained stable before increasing more than 2°C above the ambient temperature (Moran et al., 1996). Aerobic deterioration (°C) was defined as the sum of the daily temperature increases above the ambient temperature during the first 5 d of aerobic exposure (Conaghan et al., 2010).

### 2.4. Sample preparation and chemical analyses

Twenty-five grams of each sample of forage or silage was mixed with 225 mL of distilled water and blended in a Phillips Walita blender (Walita, Varginha, MG, Brazil) for 1 min at the highest setting and filtered through two layers of cheesecloth. The pH of the filtrate was measured immediately by using a pH meter (model MA522, Marconi Laboratory Equipment, Piracicaba, SP, Brazil). After the pH was measured, the filtrate was stored at −20°C for subsequent analysis of lactic acid and volatile fatty acids (acetic, propionic and butyric acids) using a high-performance liquid chromatograph (HPLC; Shimadzu model Prominence, Shimadzu Corp., Kyoto, Japan) equipped with a UV/VIS detection system and a refractive index detector (SPD-20). An apolar column (C-18 model Shimpack VP-ODS; 4.6 mm × 250 mm) was used at 35°C for chromatographic separation. The polar mobile phase consisted of a 20 mM buffer phosphate solution at pH 2.5, and acetonitrile was used as the apolar solvent. The presence of the acids was detected by UV absorbance, at a wavelength of 210 nm. Ammonia N was measured by distillation according to the AOAC (1996; method no. 941.04).

Samples of forage and silage were oven-dried (at 55°C for 72 h) and processed in a knife mill (Willey mill model 4; Arthur H. Thomas Company, Philadelphia, PA) before being ground through a 1 mm screen and analyzed for DM (105°C for 12 h) and ash (500°C for 5 h) according to the AOAC (1996; methods no., 943.01 and 924.05, respectively). Silage DM content was corrected for volatile compounds according to Weissbach (2009). Ether extract (EE) was determined according to the procedures described by the AOAC (1996; method no. 920.39). The total nitrogen (TN) was measured by rapid combustion by using a LECO Analyzer (model F528 N; LECO Corp., St. Joseph, MI, USA); crude protein (CP) was calculated as TN × 6.25. Soluble protein was determined following the procedures described by Licitra et al. (1996). The neutral detergent fiber (aNDF) and acid detergent fiber (ADF) were sequentially determined in an ANKOM^200^ Fiber Analyzer (ANKOM Technology Corporation, Fairport, NY, USA) following the procedures described by Mertens (2002) and AOAC (1990; method no. 973.18), respectively.

For aNDF analysis, a heat-stable α-amylase was used without sodium sulfite. The aNDF and ADF were expressed inclusive of residual ash. Hemicellulose was calculated as aNDF minus ADF. Neutral detergent insoluble nitrogen (NDIN) and acid detergent insoluble nitrogen (ADIN) were determined in the residual samples of aNDF and ADF, respectively, using the LECO Analyzer. Starch content of samples was determined by an enzymatic-colorimetric assay using acetate buffer in gelatinization solution and corrected for free glucose (Hall, 2009). The 30-h IVDMD, 7-h *in vitro* starch digestibility (starch-D), and 30-h *in vitro* aNDF digestibility (aNDF-D) were determined using the approach proposed by Goering and Van Soest (1970). The milk yield per tonne of DM was estimated using the Milk2006 spreadsheet (Shaver et al., 2006).

### 2.5. Statistical analyses

All silage variables were analyzed as a completely randomized design with four replicates using the MIXED procedure of SAS (v 9.2; SAS Inst. Inc., Cary, NC). The maturity stage (2 degrees of freedom (DF)), bacterial inoculation (1 DF), storage time (2 DF), and their interactions were considered fixed effects, while the residual error was considered a random effect. Differences between silage means were compared using the PDIFF option of the LSMEANS command, which is based on Fisher’s F-protected least significant difference test (multiple t-test comparisons). Similarly, when significant interactions between the factors examined occurred, means were also compared using the PDIFF option as described above. Outliers were identified and deleted if absolute values of studentized residuals exceeded ±3. Significant differences were declared at *p* ≤ 0.05. Significant differences for the main effects assessed in this study (i.e., maturity stage, bacterial inoculation, and storage time) are shown in figures only whether significant interactions between them did not occur (i.e., maturity stage × bacterial inoculation, maturity stage × storage time, bacterial inoculation × storage time, and maturity stage × bacterial inoculation × storage time); variables unaffected by treatments were only described in the text.

## 3. Results

### 3.1. Agronomic characteristics and chemical composition of whole-crop corn prior to ensiling

Whole-crop corn had a higher DM yield and percentage of grains according to the advanced maturity stage (Table 1). Corn forage harvested very early, early, and medium had 246, 306 and 353 g/kg DM, respectively. Overall, starch content increased with advances in corn plant maturity, while soluble CP, IVDMD, starch-D, and aNDF-D decreased.

**Table 1:** Characteristics of whole-corn crop prior to ensiling (data are given in g/kg DM, unless otherwise stated; mean ± SD) as influenced by maturity and bacterial inoculation^1^.

Item	Whole-crop corn at ensiling					
	Very early (250 g/kg DM)		Early (300 g/kg DM)		Medium (350 g/kg DM)	
	Control	*L. buchneri*	Control	*L. buchneri*	Control	*L. buchneri*
Forage characteristics						
DM yield, t MS/ha	13.2 ± 0.896		15.7 ± 1.23		16.9 ± 0.945	
Grains, g/kg DM	272 ± 30.6		380 ± 44.4		449 ± 56.8	
Bulk density, kg/m^3^						
Wet	750 ± 12.1	769 ± 21.6	745 ± 17.9	754 ± 14.3	709 ± 26.4	729 ± 17.2
DM	184 ± 4.82	189 ± 4.49	227 ± 5.72	232 ± 5.35	248 ± 9.07	258 ± 10.6
Nutritional value index						
pH	5.69 ± 0.080	5.66 ± 0.050	5.91 ± 0.053	6.04 ± 0.330	6.06 ± 0.105	6.22 ± 0.343
Ammonia-N, g/kg TN	46.5 ± 0.580	46.5 ± 0.420	34.1 ± 0.930	33.9 ± 0.350	28.1 ± 0.930	27.0 ± 0.570
DM, g/kg as fed	246 ± 4.54	246 ± 3.91	305 ± 4.56	307 ± 4.29	351 ± 6.79	354 ± 7.81
Ash	38.0 ± 0.900	37.5 ± 0.810	36.6 ± 1.85	36.1 ± 1.69	30.4 ± 1.91	29.7 ± 1.96
CP	109 ± 7.87	111 ± 5.49	93.6 ± 7.11	96.1 ± 14.6	83.3 ± 12.2	91.0 ± 6.44
Soluble protein, g/kg CP	403 ± 12.3	405 ± 10.1	395 ± 14.9	390 ± 9.94	355 ± 5.06	358 ± 12.6
EE	32.6 ± 0.810	33.6 ± 0.560	36.2 ± 0.320	35.6 ± 0.480	37.3 ± 0.300	37.1 ± 0.450
aNDF	483 ± 7.20	479 ± 3.40	497 ± 10.3	486 ± 10.2	516 ± 10.2	516 ± 4.77
aNDF-D, g/kg	595 ± 27.3	607 ± 22.7	541 ± 14.4	571 ± 13.2	524 ± 15.7	535 ± 19.0
ADF	247 ± 4.82	247 ± 5.59	256 ± 5.64	256 ± 5.25	271 ± 5.32	271 ± 5.44
Hemicellulose	237 ± 8.25	232 ± 8.14	241 ± 11.0	230 ± 9.91	246 ± 10.8	245 ± 5.49
Starch	158 ± 7.41	165 ± 4.96	288 ± 12.1	293 ± 7.36	353 ± 7.21	356 ± 10.0
Starch-D, g/kg	664 ± 21.2	649 ± 15.6	555 ± 6.63	588 ± 5.83	474 ± 18.2	476 ± 15.3
Digestible starch	105 ± 8.30	107 ± 5.19	164 ± 3.55	171 ± 3.89	167 ± 5.96	169 ± 5.22
IVDMD, g/kg	708 ± 12.0	724 ± 6.10	650 ± 25.5	658 ± 12.5	534 ± 12.6	552 ± 19.6
TDN_-1x_, g/kg	662 ± 16.9	677 ± 17.1	679 ± 11.2	698 ± 8.01	669 ± 9.19	674 ± 12.2
NE_L-3x_, Mcal/kg DM	1.40 ± 0.038	1.44 ± 0.033	1.48 ± 0.023	1.52 ± 0.017	1.47 ± 0.018	1.48 ± 0.021
Milk yield, kg/t DM	1,307 ± 54.2	1,354 ± 50.7	1,398 ± 33.8	1,450 ± 25.1	1,376 ± 26.9	1,386 ± 33.7

1 Corn forage was treated at ensiling either without (control) or with *Lentilactobacillus buchneri* CNCM I-4323 at 1 × 10^5^ cfu/g fresh forage.

DM, dry matter; TN, total nitrogen; CP, crude protein; EE, ether extract; aNDF, neutral detergent fiber was determined using heat-stable amylase; aNDF-D, NDF digestibility; ADF, acid detergent fiber; Starch-D, starch digestibility; IVDMD, *in vitro* dry matter digestibility; TDN_-1×_, total digestible nutrients at 1× maintenance; NE_L-3×_, net energy for lactation at 3× maintenance.

### 3.2. Fermentation, chemical composition, and aerobic stability of corn silages

**Figure 1:**
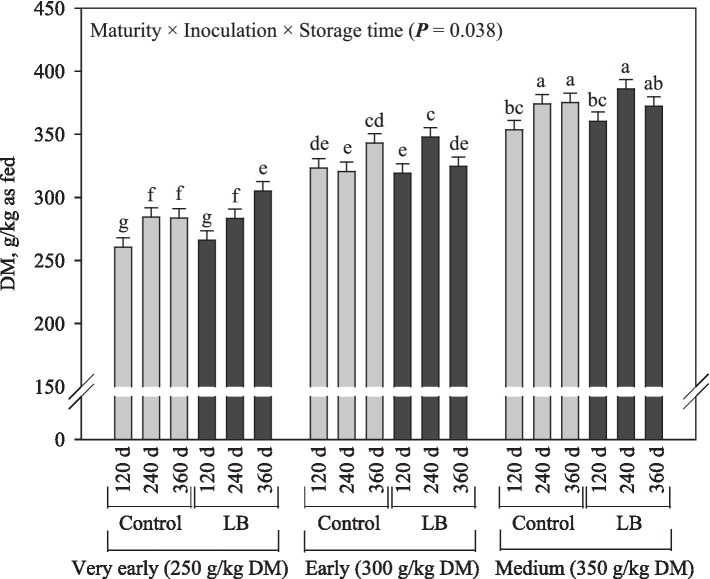
Dry matter content of corn silage as influenced by the interaction among maturity, inoculation and storage time (LB = *Lentilactobacillus buchneri*).

**Figure 2:**
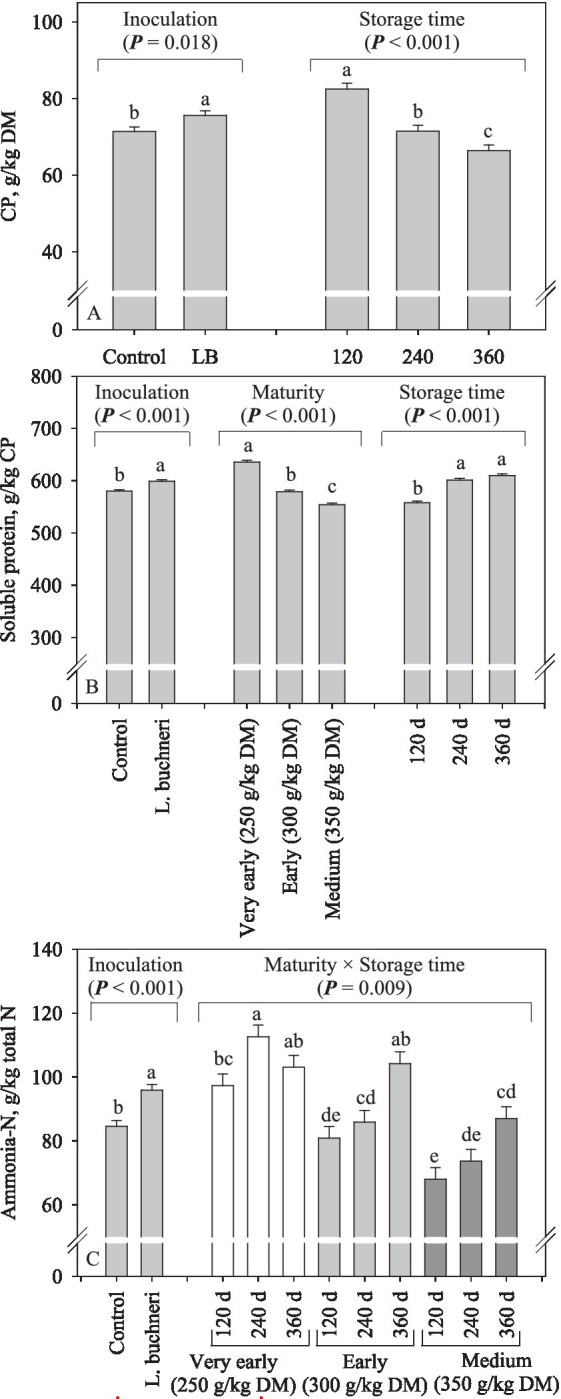
Crude protein content **(A)**, soluble CP **(B)**, and ammonia-N **(C)** of corn silage as influenced by inoculation, maturity, storage time (days), and its interaction (LB = *Lentilactobacillus buchneri*).

The DM content of corn silage was affected by the interaction between all factors assessed (Figure 1). As expected, DM content increased (*p* < 0.001) according the maturity stage advanced. With few exceptions, silages stored for 240 and 360 d had higher (*p* < 0.001) DM content than that stored for 120 d, and in general, inoculation did not increase the DM content of silages. Inoculation of corn silage with *L. buchneri* resulted in higher CP preservation (+4.2 g/kg DM; *p* = 0.018) compared to the control silage, and the CP content decreased as the storage time increased (*p* < 0.001; Figure 2A). Compared with the corn silage produced very early, the soluble CP decreased (*p* < 0.001) by 56 and 81 g/kg CP for those harvested early and at medium, respectively (Figure 2B). The soluble CP increased by 19 g/kg CP (*p* < 0.001) following silage inoculation and increased (*p* < 0.001) by 43 and 52 g/kg CP after 240 and 360 d of storage compared to the silage stored for 120 d (Figure 2B). Except for corn silages produced from plants harvested very early, increasing the storage time led to increased ammonia-N (*p* = 0.009) in the other silages; also, silage inoculation increased ammonia-N by 11.3 g/kg TN (*p* < 0.001; Figure 2C).

**Figure 3:**
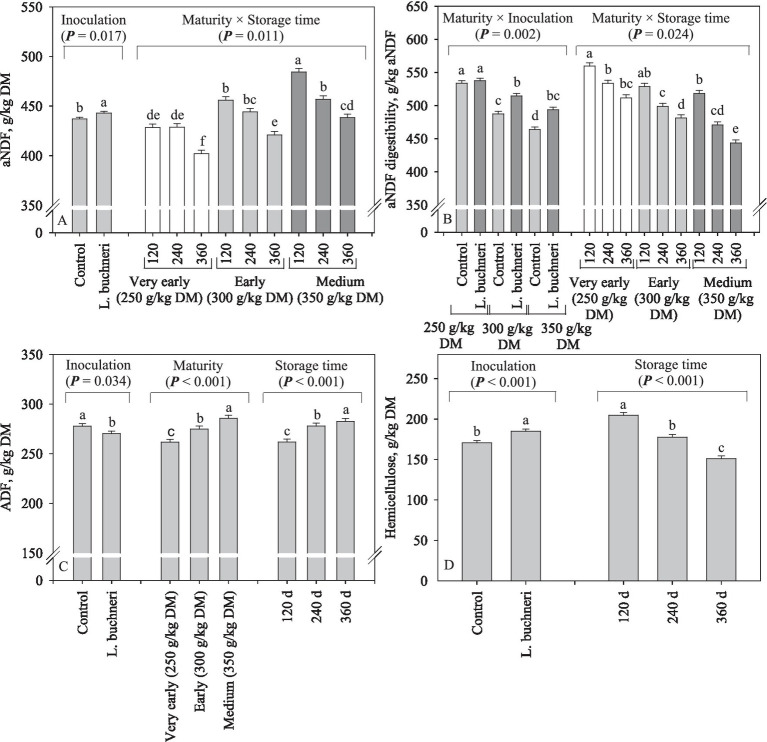
Neutral detergent fiber **(A)**, NDF digestibility **(B)**, acid detergent fiber **(C)**, and hemicellulose **(D)** content of corn silage as influenced by inoculation and interaction between maturity and storage time (days).

There was a small (6 g/kg DM) but significant (*p* = 0.017) increase in aNDF content following silage inoculation; overall, aNDF of corn silage increased by advancing plant maturity and decreased as the silages remained more time stored (*p* = 0.011; Figure 3A). Inoculation resulted in higher (*p* = 0.002) aNDF-D of corn silages produced early (+28 g/kg aNDF) and at medium (+30 g/kg aNDF), a response not observed for very early harvest; overall, aNDF-D lowered owing advances in maturity stage and storage time (*p* = 0.024; Figure 3B). Inoculation decreased the ADF content by 7 g/kg DM (*p* = 0.034), while advances in maturity and increasing storage time resulted in higher ADF content (*p* < 0.001; Figure 3C). Hemicellulose increased (*p* < 0.001) by 14 g/kg DM due to silage inoculation and decreased consistently with storage length (*p* < 0.001; Figure 3D).

**Figure 4:**
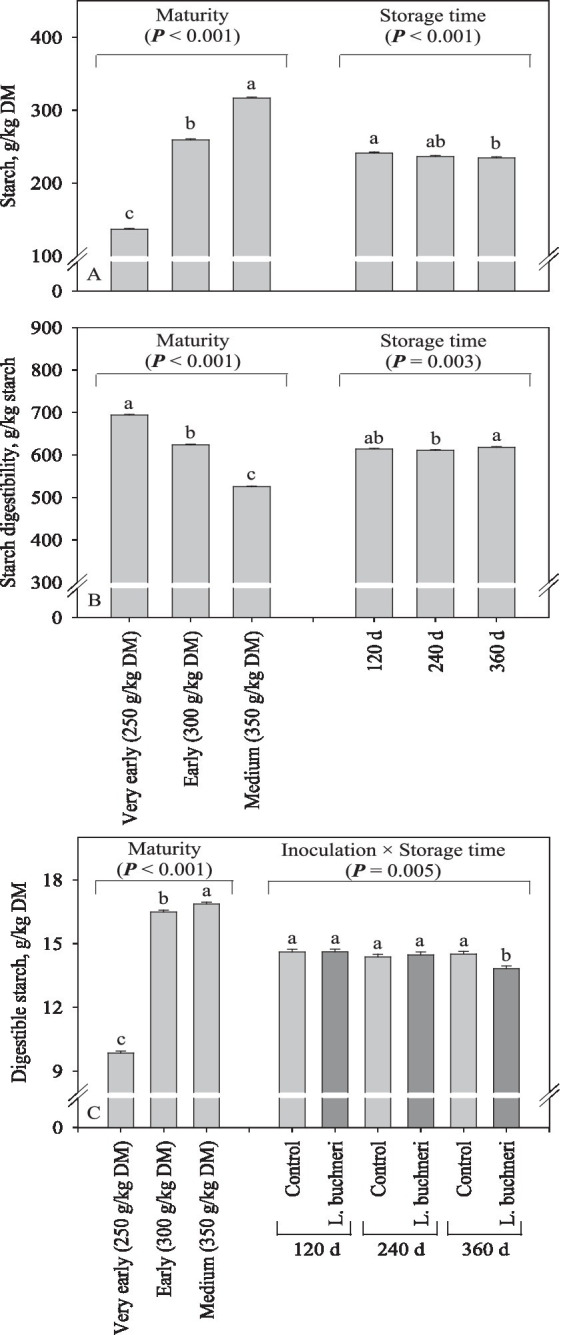
Starch content **(A)**, starch digestibility **(B)**, and digestible starch **(C)**; starch content × starch digestibility of corn silage as influenced by inoculation, maturity, storage time and its interaction.

Starch content increased (*p* < 0.001) from 137 g/kg DM in the silage produced with plants harvested very early to 317 g/kg DM in that harvested at medium (Figure 4A). Moreover, in comparison with 120 d of storage, the starch content decreased by 6 g/kg DM in corn silages stored for 360 d (*p* < 0.001). As the maturity stage advanced, starch-D decreased (*p* < 0.001) from 694 to 526 g/kg starch; there was a small increase (7 g/kg starch; *p* = 0.003) in starch-D in the silages stored for 360 d when compared to those stored for 240 d, but with no difference to 120 d (Figure 4B). The digestible starch of corn silage increased (*p* < 0.001) from 98.5 g/kg DM at the very early harvest to 169 g/kg DM at medium harvest (Figure 4C). Furthermore, the digestible starch was reduced (*p* = 0.005) by inoculation of corn silage stored for 360 d, a response not observed for the other storage times. Contents of ash (on average 41.9 g/kg DM; *p* ≥ 0.75), NIDN (on average 139 g/kg total N; *p* ≥ 0.07) and ADIN (on average 46.4 g/kg total N; *p* ≥ 0.20) were not affected by maturity stage, inoculation, or storage time.

**Figure 5:**
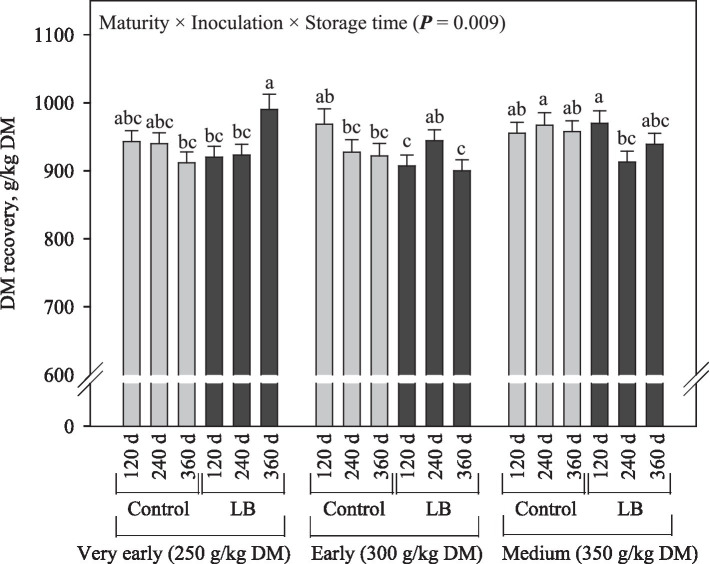
Dry matter recovery of corn silage as influenced by inoculation and the interaction between maturity and storage time (LB, *Lentilactobacillus buchneri*).

**Figure 6:**
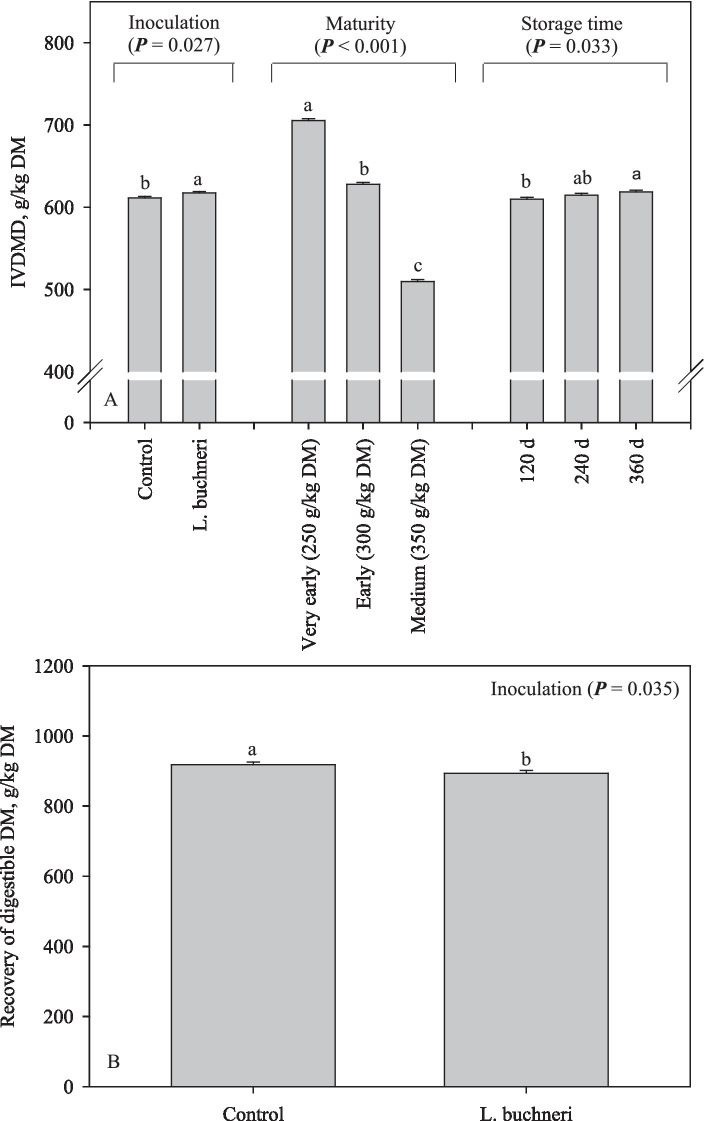
*In vitro* dry matter digestibility **(A)** and recovery of digestible DM **(B)** of corn silage as influenced by inoculation, maturity, and storage time.

There was an interaction (*p* = 0.009) between all the factors investigated in this study for DM recovery (Figure 5). However, the results were very variable depending on the maturity stage, bacterial inoculation and storage time, with no clear tendency being observed. The IVDMD slightly increased (6 g/kg DM; *p* = 0.027) due to silage inoculation and increased by 9 g/kg DM in the corn silage stored for 360 d in comparison with that stored for 120 d (*p* = 0.033); moreover, IVDMD decreased from 705 g/kg DM in the silage produced at very early harvest to 510 g/kg DM at medium harvest (*p* < 0.001; Figure 6A). The recovery of digestible DM decreased (*p* = 0.035) by 24 g/kg DM in corn silages inoculated with *L. buchneri* (Figure 6B).

**Figure 7:**
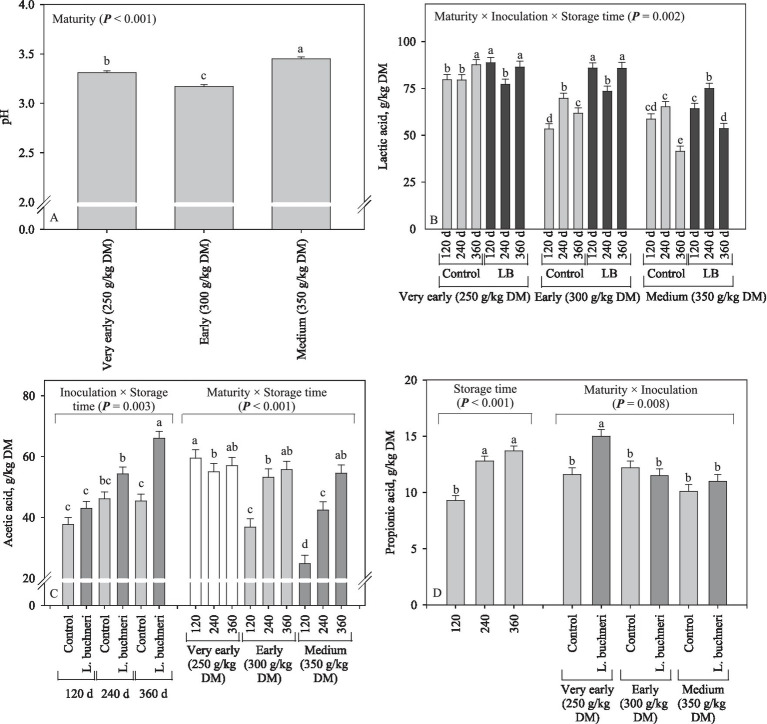
Silage pH **(A)** and concentrations of lactic **(B)**, acetic **(C)**, and propionic **(D)** acids of corn silage as influenced by inoculation, maturity, storage time (days), and its interaction.

Silage pH was only affected by maturity (*p* < 0.001), and values ranged from 3.17 (early harvest) to 3.45 (medium harvest; Figure 7A). Overall, lactic acid of corn silage decreased by harvesting more mature plants, and inoculation resulted in a higher lactic acid concentration in comparison to the control (*p* = 0.002; Figure 7B). The 2-way interaction showed that acetic acid was higher for corn silage stored for 360 d and inoculated with *L. buchneri* (*p* = 0.003); moreover, as the storage time increased, the acetic acid increased in the silages produced with more mature crops, but the silages stored for 120 d showed lower acetic acid as the maturity advanced (*p* < 0.001; Figure 7C). The concentration of propionic acid was higher (*p* < 0.001) in corn silages stored for 240 and 360 d (13.3 g/kg DM on average) in comparison with that stored for 120 d (9.30 g/kg DM); also, there was an interaction between inoculation and maturity stage, in which the inoculated silage produced at very early harvest had a higher (*p* = 0.008) concentration of propionic acid (1.51% DM) than the others (Figure 7D).

**Figure 8:**
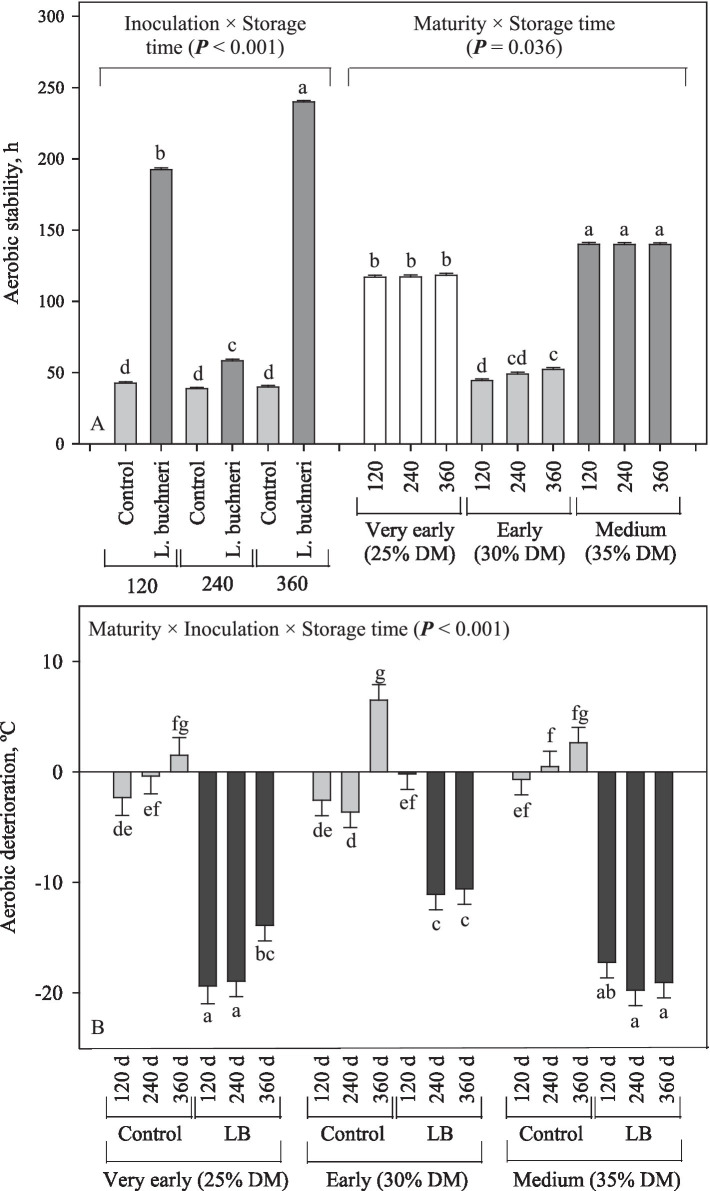
Aerobic stability **(A)** and aerobic deterioration **(B)** of corn silage during the aerobic exposure period as influenced by the different interaction ways among maturity, inoculation, and storage time.

The aerobic stability of corn silage was affected by the interaction between inoculation and storage time, in which inoculation of corn silage with *L. buchneri* persistently increased (*p* < 0.001) the aerobic stability (+123 h on average compared to the control; Figure 8A). Furthermore, the 2-way interaction (maturity × storage time) showed, in general, that more mature crop silage had higher aerobic stability (140 h; *p* = 0.036) than the others (118 and 48.5 h for those silages from very early and early harvest) and that the storage time had low impact on the aerobic stability of silages. There was a 3-way interaction for the aerobic deterioration, in which it was consistently lowered in the inoculated corn silages, except for the silage produced at early harvest and stored for 120 d (*p* < 0.001; Figure 8B).

**Figure 9:**
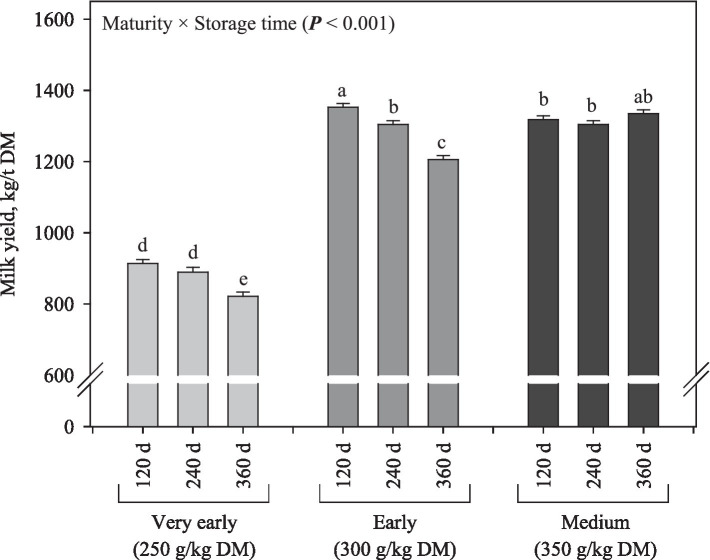
Milk yield per tonne of DM from corn silage as influenced by inoculation and interaction between maturity and fermentation length (data estimated through the Milk2006 spreadsheet).

There was an interaction between maturity and storage time for the estimated milk yield, and in general, the values increased (*p* < 0.001) as the maturity stage advanced (on average 875; 1,288 and 1,319 L/t of DM for silages produced at very early, early, and medium harvest, respectively) and decreased as the storage time was prolonged (except for the silages produced at medium harvest; Figure 9).

## 4. Discussion

In Brazil, corn silage represents the main forage source fed to dairy cows (Silva et al., 2019). As the majority of corn genotypes used for silage production in this country is flint type, strategies regarding corn silage management should focus on the improvements of starch utilization. In this regard, the current study was designed to investigate how maturity, bacterial inoculation, and storage length interact with each other in flint corn silage to obtain high-quality silage with enhanced starch-D. Agronomic features (DM yield and percentage of grains) and nutritional characteristics of the corn crop used in this study were typical of those reported under Brazilian conditions (Rabelo et al., 2015; Silva et al., 2020), with the exception of the NDF content, which had a slight increase as the maturity advanced rather than being diluted as a response of increasing percentage of grains in the whole-crop corn.

Several studies have shown that increasing storage length is a feasible strategy to enhance starch-D (Der Bedrosian et al., 2012; Windle et al., 2014; Da Silva et al., 2019). This occurs because prolamin, a protein matrix surrounding starch granules, is degraded mainly due to the proteolytic activity of bacteria and enzymes from its own plant (Junges et al., 2017). However, our results fail to support our initial hypothesis, which increasing storage time could enhance starch-D in more mature crops to a similar level as that observed in immature crop. This statement is based on the lack of an interaction between maturity and storage time for starch-D. Even though the concentration of soluble protein was similar between 240 and 360 d of storage (indicating similar proteolysis as well), corn silages stored for 360 d had a timid increase in starch-D (7 g/kg starch) in comparison to that stored for 240 d. Although significant, this response is of minor biological relevance.

The values relatively constant for starch-D likely can be attributed to the later storage interval assessed in this study (i.e., 120–360 d), which took into account only the plateau for gains in starch-D of corn silage. Our findings are in agreement with previous studies, which reported that the starch-D of both dent and flint corn silage remained constant after 45 d of storage (Der Bedrosian et al., 2012; Windle et al., 2014; Bueno et al., 2020). Indeed, broken line and segmented regression models showed that starch-D of corn silage was critically increased in the first 30 d of storage, and thereafter, the gains on starch availability were more moderate (Daniel et al., 2015; Bueno et al., 2020). Nevertheless, the ensiling process results in significant increases in starch-D, as well-documented before. For example, in this study, the starch-D of fresh forages harvested very early, early and medium were 657, 572, and 475 g/kg starch, respectively, and after 120 d of ensiling, the values increased to 696, 622, and 524 g/kg starch, respectively.

Inoculation of corn silage with *L. buchneri* increased the concentration of soluble protein, suggesting that prolamin could be degraded to a higher extent, as observed in HMC and rehydrated corn grain silage (Da Silva et al., 2018, 2019). Nevertheless, there was no effect of inoculation on the starch-D of corn silage. Moreover, the inoculated silages had increased concentrations of ammonia-N, but this variable is indicative of deamination rather proteolysis. Our results differed from those of Da Silva et al. (2018, 2019) because the material examined was different (i.e., whole-crop corn silage × corn grain silage). Prolamin represents the most abundant protein class found in corn kernels (Holding, 2014) and shifts in the bacterial community toward higher proteolytic activity likely result in greater soluble protein accompanied of improved starch-D in HMC and rehydrated corn grain silage. However, whole-crop corn has protein classes in considerable amounts other than prolamin that is present in the leaf and stalk, such as enzymatic (Rubisco and phosphoenolpyruvate carboxylase), albumin, glutelin, and extensin (Sniffen et al., 1992; Boulter and Derbyshire, 2013). Thus, an increased concentration of soluble protein in whole-crop corn silage is not necessarily associated with greater proteolysis of prolamin.

Contrary to the small changes reported by prolonging the storage time, the starch-D of corn silage was dramatically reduced by maturity (694 g/kg starch at very early to 526 g/kg starch at medium harvest). This result is not surprising because there is an increase in corn grain vitreousness as the plant becomes more mature, especially in flint hybrids (Pereira et al., 2004). The vitreous endosperm is known to be hard and crystalline, with a continuous and abundant protein matrix surrounding the starch granules (Pereira et al., 2004), which is inversely related to ruminal starch disponibilization. A similar reduction in starch-D was observed by Der Bedrosian et al. (2012) working with normal and brown midrib hybrids harvested at 320 and 410 g/kg DM. However, as the whole-crop corn became more mature, the starch content and amount of digestible starch increased considerably. This result is particularly important because the starch accumulation may lead to the reduction of concentrate utilization inside the farm and the increased amount of digestible starch might result in increased milk yield.

It was also noted that advances in maturity increased aNDF content and decreased aNDF-D, which was expected. As the plant matures, there is an increase in fiber content accompanied by higher lignification of the cell wall, which is indigestible and therefore harmful fiber digestion.

Although the results from the literature are controversial, increasing the storage length was proposed to enhance NDF-D (Hallada et al., 2008). However, in the current study the aNDF-D was consistently reduced across the maturities examined as the storage increased. This finding is associated with the acid hydrolysis of hemicellulose, caused mainly by the acidic environment within the silo as a consequence of fermentation (McDonald et al., 1991). This means that the hemicellulose disappearance led proportionally to an increase in the concentration of the indigestible fiber fraction in corn silages stored for a longer time, explaining the lower aNDF-D.

Furthermore, the aNDF-D increased following the bacterial inoculation of silages produced at early and medium harvest. The strain of *L. buchneri* used in this study was not assessed regarding its capacity to produce ferulic acid esterase (FAE). Nevertheless, it is well recognized that some strains of *L. buchneri* can produce FAE (Nsereko et al., 2008; Addah et al., 2012), an enzyme that usually leads to improved fiber digestion by releasing ferulic acid from cell-wall arabinoxylans and then increasing its susceptibility to microbial attachment (Kang et al., 2009). However, further studies are needed to confirm the hypothesis of FAE production by *L. buchneri* CNCM I-4323, since actually it is only a speculation because it was not measured. Additionally, inoculation of corn silage resulted in higher IVDMD, probably due to the increased aNDF-D found in this silage.

The inoculation of corn silage with *L. buchneri* increased the concentration of acetic acid in the silage produced from medium harvest. *Lentilactobacillus buchneri* is a heterofermentative lactic acid bacteria (LAB) that, under anaerobic conditions, metabolizes some quantity of lactic acid into acetic acid and other products and then, inoculated silages usually have lower concentrations of lactic acid and higher acetic acid (Bernardi et al., 2019). However, in general, the inoculated silages had similar concentrations of lactic acid as observed in the control, but the causes for that are unclear.

The utilization of heterofermentative LAB such as *L. buchneri* often leads to increased DM loss in corn silage (Bernardi et al., 2019). This occurs because there is CO_2_ production in the heterofermentative pathway. In the current study, there was a 3-way interaction for DM recovery, but in general, inoculation did not decrease DM recovery of corn silage as expected, likely because the lactic acid was not lowered. Moreover, all the silages had good DM recovery (i.e., > 900 g/kg DM). However, the recovery of digestible DM was slightly reduced by inoculation.

Harvesting more mature whole-crop corn resulted in a less intense fermentation, which can be seen by the lower concentrations, in general, of lactic and acetic acid for the corn silage produced with 350 g/kg DM. This occurs because increasing maturity leads to the reduction of water activity that is available for the metabolism of microorganisms, and then, the growth of microorganisms is depressed (McDonald et al., 1991). Moreover, silages produced at early and medium harvest had increased acetic acid concentrations as the storage time increased. This indicates that in longer storage periods, the population of heterofermentative bacteria probably plays a more important role in the fermentation process because it remains fairly active (McDonald et al., 1991; Kleinschmit and Kung, 2006). Despite increases in acetic acid as storage increased, there was no improvement in the aerobic stability of corn silage stored for a longer time. It is known that well-fermented corn silage usually has low aerobic stability under tropical conditions because elevated temperatures favor the growth of yeasts (Daniel et al., 2019), which initiate the aerobic deterioration of silage by using lactic acid as a substrate. In this regard, *L. buchneri* has been successfully used to improve the aerobic stability of silages (Bernardi et al., 2019), which was confirmed in this study. This bacterium inhibits the growth of yeasts and molds by increasing the acetic acid concentration in the silage, which has antifungal properties (Driehuis et al., 1999; Danner et al., 2003; Kleinschmit and Kung, 2006) and then, decrease the aerobic deterioration of silage, as observed in the current study.

In this study, the estimated milk yield obtained through the Milk2006 spreadsheet (Shaver et al., 2006) was most directed toward determining the ideal range of maturity stages in which whole-crop corn should be harvested. This statement is based on two points: (1) the Milk2006 spreadsheet did not consider the improvements in aerobic stability from bacterial inoculation and the consequent reduction in silage spoilage after the silos are opened, recognizably as the main benefit of using *L. buchneri*; and (2) the Milk2006 spreadsheet did not take into account starch-D. The lower milk yield observed as the storage time increased can be attributed to the reductions in CP content and aNDF-D. For the principal purpose of using the Milk2006 spreadsheet, milk yield was significantly increased (on average, +428 L/t of DM) when silages were produced at early and medium harvest compared to those produced at very early harvest. This result can be attributed mainly to the starch accumulation in corn silages produced with plants harvested later. It is worth noting that our findings meet the recommendation of DM content ranging from 300 to 350 g/kg for whole-crop corn harvest in tropical conditions when pull-type forage harvesters are used in the process (Jobim et al., 2007; Oliveira et al., 2017; Daniel et al., 2019). This recommendation is based on (1) agronomic advantages such as increased DM yield and percentage of grains, (2) benefits for the ensiling process, since a lower amount of water is transported from the field to the silo and DM loss through effluent production is reduced, (3) benefits to the fermentation process, considering that the growth of undesirable microorganisms (e.g., Clostridia and Bacilli) is avoided or at least impaired by increasing DM content of forage, and (4) nutritional advantages such as higher starch accumulation in the grain.

## 5. Conclusion

The storage for a longer time (i.e., >120 d) with the goal of increasing silage digestibility did not occur. Despite reducing silage digestibility and based on our experimental conditions, this study showed that harvesting whole-crop flint corn with 300 to 350 g/kg DM is desirable to have higher DM yield and starch accumulation, which lead to increased milk yield. Inoculation with *L. buchneri* is necessary to preserve the silage against aerobic deterioration.

## Data availability statement

The raw data supporting the conclusions of this article will be made available by the authors, without undue reservation.

## Ethics statement

The animal study was reviewed and approved by all procedures adopted in this study were performed according to Ethical Principles in Animal Experimentation from the National Council for Animal Experiment Control (CONCEA) and were approved by the Ethics Committee on the Use of Animals (CEUA) from São Paulo State University (UNESP) at a regular meeting (Protocol No. 006764/17). Written informed consent was obtained from the owners for the participation of their animals in this study.

## Author contributions

RR, GS, and EV conceived and designed the research. LR, MA, MS, and CR conducted the experiment. RR, CR, and WS analyzed the data. LR, RR, CR, and WS wrote the manuscript. All authors contributed to the article and approved the submitted version.

## Funding

This work was funded by the São Paulo Research Foundation (FAPESP grants #2017/00696–6, #2018/21568–9, and #2016/00446–7; São Paulo, SP, Brazil), and the National Council for Scientific and Technological Development (CNPq grant #142190/2016–0; Brasília, Brazil) for providing a scholarship.

## Conflict of interest

The authors declare that the research was conducted in the absence of any commercial or financial relationships that could be construed as a potential conflict of interest.

## Publisher’s note

All claims expressed in this article are solely those of the authors and do not necessarily represent those of their affiliated organizations, or those of the publisher, the editors and the reviewers. Any product that may be evaluated in this article, or claim that may be made by its manufacturer, is not guaranteed or endorsed by the publisher.
